# Changes in perceived knowledge about childbirth among pregnant women participating in the Senses of Birth intervention in Brazil: a cross-sectional study

**DOI:** 10.1186/s12884-020-02874-3

**Published:** 2020-05-05

**Authors:** Luísa M. M. Fernandes, Sônia Lansky, Bernardo J. Oliveira, Amélia A. L. Friche, Christine T. Bozlak, Benjamin A. Shaw

**Affiliations:** 1 Department of Health Policy, Management, and Behavior, School of Public Health, University at Albany, State University of New York, One University Place, Rensselaer, NY 12144 USA; 2 Department of Health, City Hall, Belo Horizonte, Minas Gerais Brazil; 3 School of Education, Universidade Federal de Minas Gerais, Belo Horizonte, Brazil; 4 School of Medicine, Universidade Federal de Minas Gerais, Belo Horizonte, Brazil

**Keywords:** Maternal health, Childbirth, Health education, Cesarean section, Evidence-based medicine, Women’s knowledge

## Abstract

**Background:**

Senses of Birth (SoB) is a health education intervention in Brazil that aims to reduce unnecessary cesareans in the country by providing information on reproductive rights, benefits and risks of childbirth, and use of intrapartum evidence-based practices (EBP) which are recommended by the World Health Organization (WHO) to improve childbirth outcomes and satisfaction. This study evaluates the impact of the SoB on pregnant women’s perceived knowledge about normal birth (NB), cesarean, and use of EBP.

**Methods:**

1287 pregnant women answered a structured survey immediately after their visit to the intervention, between March 2015 and March 2016. To estimate the potential impact of the intervention on women’s perceived knowledge, and possible associations between sociodemographic characteristics and perceived knowledge, statistical analyses were performed, including paired T-tests, ANOVA, and logistic and linear regressions.

**Results:**

The mean score (MS) of perceived knowledge after the intervention was higher than the MS before experiencing the intervention for all three knowledge domains: Normal Birth (MS Before = 3.71 x MS After = 4.49), Cesarean (MS Before = 3.54 x MS After = 4.26) and EBPs (MS Before = 3.14 x MS After = 4.14). The results suggest that perceived knowledge increased more for low-income women (B = 0.206; *p* < 0.001 for EBP), women without private health insurance (OR 2.47, 95% CI: 1.49–4.09 for NB), with private prenatal care (OR 2.42, 95% CI: 1.59–3.66 for NB), experiencing their first pregnancy (OR 1.92, 95% CI: 1.31–2.82 for EBP; OR 1.37, 95% CI: 1.03–1.84 for NB; OR 1.37, 95% CI: 1.03–1.84 for cesarean), and in their first or second trimester (OR 1.64, 95% CI: 1.13–2.39 for EBP; OR 1.48, 95% CI: 1.11–1.97 for NB; OR 1.85, 95% CI: 1.40–2.41 for cesarean).

**Conclusion:**

The study showed that participation in the SoB was associated with an increase in perceived knowledge among Brazilian pregnant women. The intervention gains relevance considering the lack of evidence of the impact of non-clinical interventions to reduce unnecessary cesareans in middle and low-income countries.

## Background

Since 1985, the World Health Organization (WHO) has recommended that C-section rates should be between 10 to 15% of live births worldwide [1]. Medically-indicated cesareans are effective in preventing maternal and infant morbidity and mortality. However, several studies have shown that C-section rates higher than 15% are associated with an increase in maternal mortality and morbidity, including a higher risk of a prolonged hospital stay, hysterectomy caused by postpartum hemorrhage, postnatal treatment with antibiotics and cardiac arrest for women, among adverse birth outcomes [1–6].

In Latin America, the average C-section rate is 33 per 100 live births, 49% of which are considered elective [5, 7]. Causes of the higher rates are multifactorial, including socio-inequalities, cultural preferences, clinical recommendations not based on the best scientific evidence, underuse of evidence-based practices, practice of defensive medicine, lack of midwife availability/support, value/cost of medical procedures (economical interest), increased use of technology, and increased medicalization of childbirth [8–13].

Countries with alarming cesarean rates, such as Brazil, Dominican Republic, Egypt, Taiwan, China, India, and Iran, have many similar non-clinical factors identified as contributors to the high rates, as well as unique factors to each country’s context [13–18]. Researchers found that social and cultural beliefs of women, families, and communities in these countries result in a similar viewpoint that a cesarean is a safer delivery mode for the mother and child when compared to vaginal birth. More specifically, common perceptions in these countries include: women are not physically and mentally prepared for vaginal childbirth, vaginal birth can impact a woman’s future sexual life, medical interventions are standard unavoidable practices for vaginal births, and the process of birth is unimportant [10, 19–21].

United Nations (UN) Sustainable Development Goals (SDG) call for a new era of accountability, challenging the health systems to identify and eliminate the preventable maternal morbidity and mortality associated with inadequate access to services, or the delivery of services that are “too little, too late” (TLTL) [22–24]. The SDGs also question the opposite extreme reality of the over-medicalization of regular antenatal, intrapartum, and postnatal care, referred to as “too much, too soon” (TMTS) [22–24].

SDG #3 - to reduce global maternal mortality to less than 70 per 1000 live births without any country with double of the global average, and SDG #5 - gender equality – ensuring access to reproductive health and reproductive rights [25] can be addressed through the lens of the new WHO recommendations for intrapartum care [26]. WHO presents a comprehensive document that creates a platform for pregnant women with respectful, individualized, woman-centered, and effective clinical and non-clinical practices to optimize birth outcomes for the woman and her baby [24, 26]. The protocol reinforces that evidence-based practices can be effective strategies for both scenarios – TMTS and TLTL – to ensure women’s reproductive rights [23].

The Brazilian maternity health care system is mostly interventionist, an example of a “too much, too soon” approach where labor and childbirth are considered a medical event instead of a normal physiologic process with its own social and cultural context. Brazil’s C-section rate was 55.7% in 2017 [27]. Rates have been increasing since the year 2001 and by 2009 exceeded the number of vaginal deliveries. C-section rates in Brazil reached 57% in 2014 [28–30], of which 47.2% were classified as unnecessary [31]. Fifty-eight percent of births in Brazil happen in the private sector, among which 83% of the deliveries are cesareans, while in the public sector, the C-section rate is 40% [32, 33].

Additional reasons for the high cesarean rate in Brazil might be related to the model of care and physicians’ beliefs and behavior. Different authors have discussed the increased rates of cesarean in the private sector, creating a paradox of care, in which low-risk women have more interventions [34–36]. Studies show that, frequently, the relationship between women and obstetricians is asymmetrical and physician-centered, with no place for women’s choices and preferences [16, 36, 37]. Therefore, Brazil’s maternal health care model is considered a technocratic model of childbirth [38, 39].

The technocratic model of childbirth is the hegemonic model around the world, centered on the physician’s authoritative knowledge, hospital procedures, and technology [40–42]. On the other hand, the humanistic model emphasizes the connection between the mind and body, focusing on a flexible approach and balance between the need of the institution and individual/tailored continuum of care [40, 41]. In Brazil, the humanistic model has been advocated by community-based movements since the beginning of the 1990s [42], and concepts of the model have been incorporated into public policies, such as the most recent “Stork Network”, a public health program aiming to implement the best practices in childbirth in Brazil [43]. However, the changes were not sufficient to prevent a TMTS scenario, with practices firmly anchored by authoritative medical knowledge.

Authoritative knowledge is a set of scientific-based information more commonly available to physicians and other healthcare professionals, and embodied knowledge is based on the individual’s perception/intuition and practical experience [44, 45]. The knowledge set a woman has, embodied, authoritative, or a combination of both is a distinct domain of control, and control is often linked to a broad notion of “good” birth [11]. Women’s perceptions of birth and decisions for type of birth are a combination of embodied knowledge and authoritative knowledge since the technocratic and the humanistic models coexist in most maternal care models around the world [21, 40, 45]. Women might exercise control of the body during childbirth with use of EBP, such as pain management (non-pharmacological methods), feeling supported or cared for (one-to-one support/companionship), and active, informed consent, including respect of her wishes by the health professional attending the childbirth (birth plan) [11].

Although using EBPs during labor and childbirth is recommended to improve birth outcomes [46–51], they are still underutilized practices [38, 52–55], while the poor maternal mortality and morbidity rates indicate that their implementation still needs to be reinforced [56–58]. EBPs during labor and childbirth, also known as best practices, can be effective strategies for both scenarios TMTS and TLTL, and to ensure women’s reproductive rights [23]. Between 2010 and 2015, there were 163 childbirth guidelines published around the world that recommended different EBP to promote respectful care, increased communication, ensure appropriate informed consent, guarantee that reproductive rights are respected, and support a positive childbirth experience [23].

Considering the urgent need to reduce unnecessary cesareans in Brazil and the multifactorial reasons for the increasing rates, a health education and health promotion intervention named Senses of Birth (SoB) was implemented in Brazil [59]. This study evaluates the impact of the SoB intervention on pregnant women’s perceived knowledge about normal birth, cesarean, and the use of evidence-based practices during labor and childbirth.

## Methods

The study has a cross-sectional design, with a quantitative analysis of the impact of the Senses of Birth intervention on pregnant women’s perceived knowledge about the type of birth and the use of evidence-based practices during childbirth. It is part of the research project named “Senses of Birth: Effects of the interactive intervention in changing perceptions on labor and childbirth,” funded by the Bill and Melinda Gates Foundation, approved by the Federal University at Minas Gerais IRB (COEP/UFMG, 934.472) and by the University at Albany Institutional Review Board (IRB Protocol Number: 18-X-209-01).

### The health education intervention

SoB is an interactive intervention where visitors (women, men, children, adolescents) are invited to walk through the pregnancy and childbirth process, first as a pregnant woman and later as the newborn [59]. During the experience, the visitor engages in themes related to normal birth, risks of cesarean, best practices during childbirth, obstetric violence, the Ministry of Health (MS), and WHO recommendations, and the Brazilian humanization movement [59].

The SoB intervention was assembled in public spaces, such as parks, shopping malls, and university campuses, to reach a diverse group of people and invite everyone to join the experience. Aiming to inform, engage, and arouse emotions, SoB was structured as an immersive experience, raising the visitors’ awareness of the need to improve labor and childbirth care. The intervention design promotes the interaction between the visitors and intervention. It combines digital art, theatrical techniques, and media (video, photography, sets, written panels) to provide the visitors different sensations while walking through the intervention.

First, the visitors see themselves becoming pregnant, where a sensor captures the person’s image and projects a pregnancy development. In the sequence, as a pregnant person, the visitor can shop for childbirth products [59–61]. The market is a parody on the contemporary tendency of consumerism and immediacy due to the practicality and commodification of childbirth [59–61]. Examples of typical practices in Brazil associated with childbirth and products sold at the market are: Scheduling a C-section; photographers; beauty salon services; a party in the hospital room after the C-section; and artificial milk [59–61].

The next part of the intervention invites visitors to watch the dynamic dialogue between a pregnant woman, a mother with a newborn, a friend, an obstetrician, a doula, and a midwife [59–61]. The dialogue brings to light myths about birth, accurate information, and data to generate questions regarding expectations for the birth [59–61]. Finally, the visitor is invited to end the journey as a baby on the path to being born [59–61]. The group walks through the womb and vaginal canal with sensory stimulation, creating an individual emotional experience [59–61].

The visit is 20–40 min long, and after that, all are invited to see the pictures, movies, and join a group discussion regarding the approached topics [59–61]. The group discussions engage the local community and grassroots movements that often bring speakers and provide support for the discussion around the themes approached at the intervention [59–61]. The intervention’s website and social media are used to maintain a continuous source of information for anyone interested [59–61].

SoB used the Theory of Planned Behavior (TPB) to support the development of a culturally appropriate intervention and to ground data collection tools developed for the project evaluation [42–44]. TPB is used to understand determinants of behavior over which individuals do not exert complete control [49], and in this case was used to support the understanding of pregnant women’s increasing knowledge after participating at SoB and the use of EBP during labor and childbirth. According to TPB, behavior can be directly influenced by the intention to engage in that behavior [49, 50]. The intention to perform a behavior is a function of one’s attitudes, subjective norms, and perceived control over the behavior [49, 51].

Attitudes reflect someone’s personal opinion, or the value attributed to the behavior, that is if the behavior is good or bad or can bring positive or negative outcomes [62, 63]. An opinion about behavior can be formed based on experiences, examples, and knowledge regarding the possible outcomes [62, 63]. Subjective norms refer to the social expectations and desires around a specific behavior, and how motivated is the person to adhere to those expectations [62, 63]. The perceived behavioral control is primarily the individual’s perceived self-efficacy, that is the confidence in their ability to perform the behavior [49, 51]. Such perception of control is built by internal factors (knowledge acquired, and skills learned), and external factors (practical resources available, opportunities to use them, and the presence of other supportive conditions) [62, 63].

Women’s attitudes regarding childbirth and use of EBP are then formed by the amount of appropriate information regarding risks and benefits they have, as well as their previous experience and/or examples from their communities. A positive attitude, therefore, is a belief that using an intrapartum EBP will contribute to a favorable or desirable outcome, and this type of attitude can positively impact one’s decision to use the practices. For a woman to perceive that she can control a behavior, such as using the intrapartum EBP, she will also need knowledge, skills, resources, and opportunities to increase her self-efficacy. Thus, this study focused on assessing the extent to which participation in the SoB may have influenced women’s knowledge regarding birthing options and EBP.

### Data and sample

The developers of the SoB intervention used the TPB to ensure the intervention was culturally appropriate and to ground data collection tools developed for the evaluation of the intervention [59–61]. The self-administered survey included questions that asked about the women’s changes in perceptions, feelings, preferences, and perceived knowledge related to normal birth, cesarean, and evidence-based practices, before and after visiting the Senses of Birth intervention. Socioeconomic and demographic characteristics and experiences with previous and current pregnancies were also collected using the survey.

Twenty-two thousand six hundred and twenty-one (22,621) people visited the SoB intervention during the data collection period, and all pregnant women over 18 years old were invited to participate in the intervention evaluation [59]. Using the convenience sampling method, one thousand two hundred and eighty-seven (1287) pregnant women answered a structured survey, supported by locally trained interviewers available to answer questions. The number of interviewers on-site varied, considering the city and flux of participants throughout the day. Women answered the self-applied survey between March 2015 and March 2016, in five different cities of Brazil (Belo Horizonte/MG; Rio de Janeiro/RJ and Niterói/RJ; Ceilândia/DF, and Brasília/DF) and provided written informed consent. The survey was administered at one point in time, after participating in the intervention, to avoid influencing the visitor’s experience.

### Measures

Three groups of variables were selected for analysis. They were categorized or scored for analysis in this study as follows:

1. Socio-demographic characteristics. Participants were asked to report their: age (19–34 years and ≥ 35 years), education (≤ 12 years, > 12 years), and private health insurance (yes, no). Private health insurances in Brazil are health care plans privately or employer funded. Income was measured using the monthly family earning measure by the country minimum wage (< 2 minimum wages (MW), 2 to < 5 MW, and 5 to < 10 MW, and ≥ 10 MW). One minimum wage at the time of the intervention was approximately U$224.14, and the federal government annually defines the value. Race was self-reported and categorized as white, black (pardo/black), or other (Asian and indigenous).
2. Pregnancy Information. Participants were asked about the following topics regarding their pregnancy: public (SUS public health system) or private (Private Health Insurance and/or Out of Pocket payment) prenatal care coverage, gestational age when visiting the intervention (1st and 2nd trimester, 3rd trimester), and first pregnancy (yes, no).
3. Perceived Knowledge. Participants were asked to self-report their perceived knowledge, before (retrospectively) and after the intervention, about: normal birth; cesarean; risks of normal birth; risks of cesarean; doula support; midwife care; right to have companionship of her choice throughout the hospital stay, during labor and childbirth; access to non-pharmacological birth pain relief methods; birth plan; childbirth best practices; social organizations that advocate for humanized and evidence-based care; Brazil’s C-section rate; the Ministry of Health (MS) and World Health Organization (WHO) guidelines for labor and childbirth care; and obstetric violence. Answers were chosen from a Likert scale, to increase the accuracy and control for possible confirmation bias, ranging from none (1); poor (2); fair (3); good (4); and very good (5). For example, the perceived knowledge about normal birth was measured using the following two questions:

- Your knowledge about NORMAL BIRTH BEFORE the intervention was: (1) None; (2) Poor; (3) Fair; (4) Good; (5) Very good.
- Your knowledge about NORMAL BIRTH AFTER the intervention was: (1) None; (2) Poor; (3) Fair; (4) Good; (5) Very good.

### Preparing variables

A factor analysis using principal domain analysis as the extraction method and the varimax rotation with Kaiser normalization was performed to identify clusters of perceived knowledge variables with shared variance. Three clusters, named here as domains, were identified. Domain 1 included eleven perceived knowledge variables focused on Evidence-based Practice (EBP), including doula support; midwife care; right to have a companionship of her choice throughout the hospital stay, during labor and childbirth; access to non-pharmacological birth pain relief methods; birth plan; childbirth best practices; organizations that defend the humanized and evidence-based care; Brazil’s C-section rates; MS and WHO guidelines for labor and childbirth care; obstetric violence. Domain 2 included two perceived knowledge variables focused on Normal Birth (NB), including the variables “perceived knowledge about normal birth” and “perceived knowledge about risks of normal birth.” Domain 3 included two perceived knowledge variables focused on Cesarean Knowledge, including the variables “perceived knowledge about cesarean” and “perceived knowledge about risks of cesarean.”

The continuous variables ranging from 1 to 5 points were combined to create two mean scores for each of the three domains: one mean score for perceived knowledge before exposure to the SoB intervention, and one for the mean score after exposure to the SoB intervention. Subsequently, the mean score variables were used to create change scores by subtracting the mean score after from the mean score before the intervention, resulting in a continuous variable for each domain ranging from − 5 to 5.

For further analysis, the change score variables were transformed into dichotomous variables representing an *increase* or *no increase* in perceived knowledge. Women who presented a change score from 0.1 to 5 were classified as “*perceived increase in knowledge after the intervention*,” while women with a change score between − 5 and 0 were grouped as “*no perceived increase in knowledge after the intervention*.”

### Analysis

Associations between social-demographic (SD) and pregnancy information (PI) variables were analyzed with chi-square tests to identify differences between groups that changed and did not change perceived knowledge before and after the SoB intervention for each of the three knowledge domains. ANOVA tests were performed to identify associations between SD and PI variables and amounts of change in the mean perceived knowledge score before the intervention. All scores presented normal distribution. A paired T-test was used to compare the mean perceived knowledge score before with the mean perceived knowledge score after the intervention for each domain.

Multivariate analyses also assessed these associations. A logistic regression model was used to identify variables that were independently associated with “increased perceived knowledge” after the intervention. A linear regression was performed to analyze the independent variables associated with amounts of change in perceived knowledge for each domain. All selected variables were kept into the regression models regardless of the previous statistical association, considering the comparability between models and the literature support of the variables. The magnitude of associations in the logistic regression models was evaluated through odds ratio (OR) and their respective confidence intervals at 95%. Model adjustments were evaluated using the Hosmer and Lemeshow test. The magnitude of associations in the linear regression models was evaluated using the Beta values, with statistical significance set at alpha = .05. Model adjustments were evaluated using the R-square test, and multicollinearity was tested, and the residual analysis performed. The statistical program IBM SPSS Statistics 24R was used for the data analysis.

## Results

Pregnant women who participated in this study were predominantly 19 to 34 years old (81.8%), black (54.2%), with more than 13 years of education (68.4%), had a family monthly income between 2 to < 5 minimum wages (MW) (32.2%), were primiparous (50.9%), in the first or second trimester on the day of their visit to the intervention (60.9%), had private health insurance (74.7%), and private prenatal care coverage (65.0%) (Table 1).

**Table 1:** Socio-demographic characteristics and pregnancy information of pregnant women related to perceived knowledge change after the intervention

		EBP Knowledge Domain			Normal Birth Knowledge Domain			Cesarean Knowledge Domain		
Characteristics	Total^b^	Increase	Non-Increase	Chi-square (*P*-value)	Increase	Non-Increase	Chi-square (*P*-value)	Increase	Non-Increase	Chi-square (*P*-value)
	N (%)	N (%)	N (%)		N (%)	N (%)		N (%)	N (%)	
Age										
19–34 years	1043 (81.8)	891 (82.2)	152 (79.6)	0.337	727 (83.0)	316 (79.2)	0.095^^^	669 (82.6)	374 (80.4)	0.495
≥ 35 years	232 (18.2)	193 (17.8)	39 (20.4)		149 (17.0)	83 (20.8)		141 (17.4)	91(19.6)	
TOTAL	1275	1084 (85.0)	191 (15.0)		876 (68.7)	399 (31.3)		810 (63.5)	465 (36.5)	
Education										
≤ 12 years	398 (31.6)	352 (32.8)	46 (24.7)	0.006^*^	309 (35.9)	89 (22.4)	0.000^**^	268 (33.4)	130 (28.5)	0.063^^^
> 12 years	869 (68.4)	720 (67.2)	140 (75.3)		552 (64.1)	308 (77.6)		534 (66.6)	326 (71.5)	
TOTAL	1258	1072 (85.2)	186 (14.8)		861 (68.4)	397 (31.6)		802 (63.8)	456 (36.2)	
Income^a^										
< 2 MW	282 (23.9)	249 (24.7)	33 (19.2)	0.001^**^	218 (23.9)	64 (17.3)	0.000^**^	185 (24.7)	97 (22.5)	0.002^*^
2 to < 5 MW	380 (32.2)	337 (33.5)	43 (25.0)		279 (32.2)	101 (27.3)		258 (34.5)	122 (28.3)	
5 to < 10 MW	293 (24.9)	242 (24.0)	51 (29.7)		193 (24.9)	100 (27.0)		185 (24.7)	108 (25.1)	
≥ 10 MW	224 (19.0)	179 (17.8)	45 (26.2)		119 (19.0)	105 (28.4)		120 (16.0)	104 (24.1)	
TOTAL	1179	1007 (85.4)	172 (14.6)		809 (68.6)	370 (31.4)		748 (63.6)	431 (36.6)	
Race										
White	587 (45.9)	494 (45.4)	93 (48.4)	0.247	385 (43.8)	202 (50.4)	0.035^*-^	355 (43.6)	232 (49.8)	0.026^*^
Black and Others	691 (54.2)	594 (54.6)	99 (51.6)		494 (56.2)	199 (49.6)		459 (56.4)	234 (50.2)	
TOTAL	1280	1088 (85.0)	192 (15.0)		879 (68.7)	401 (31.3)		814 (63.6)	466 (36.4)	
First Pregnancy										
Yes	593 (50.9)	506 (51.3)	65 (36.5)	0.020^*^	401 (51.0)	170 (45.1)	0.409	390 (53.1)	181 (42.1)	0.001^**^
No	571 (49.1)	480 (48.7)	113 (63.5)		386 (49.0)	207 (54.9)		344 (46.9)	249 (57.9)	
TOTAL	1164	986 (84.7)	178 (15.3)		787 (67.6)	377 (32.4)		734 (63.1)	430 (36.9)	
Private Health Insurance										
Yes	958 (74.7)	810 (74.3)	148 (77.1)	0.253	623 (71.0)	335 (82.9)	0.000^**^	600 (73.5)	358 (76.8)	0.155
No	324 (24.3)	280 (25.7)	44 (22.9)		255 (29.0)	69 (17.1)		216 (26.5)	108 (23.2)	
TOTAL	1282	1090 (85.0)	192 (15.0)		878 (68.5)	404 (31.5)		816 (63.7)	466 (36.3)	
Prenatal Care Coverage										
SUS^c^ (Public)	439 (35.0)	374 (35.1)	65 (34.8)	0.982	312 (36.2)	127 (32.5)	0.202	282 (35.2)	157 (34.7)	0.853
Private	814 (65.0)	692 (64.9)	112 (65.2)		550 (63.8)	264 (67.5)		519 (64.8)	295 (65.3)	
TOTAL	1253	1066 (85.1)	187 (14.9)		862 (68.8)	391 (31.2)		801 (63.9)	452 (36.1)	
Pregnancy Trimester at the time of the intervention										
1st or 2nd	732 (60.9)	637 (62.3)	95 (53.1)	0.016^*^	519 (63.7)	213 (55.0)	0.003^*^	497 (65.6)	235 (52.9)	0.000^**^
3rd	470 (39.1)	386 (37.7)	84 (46.9)		296 (36.3)	174 (45.0)		261 (34.4)	209 (47.1)	
TOTAL	1202	1023 (85.1)	179 (14.9)		815 (67.8)	387 (32.2)		758 (63.1)	444 (36.9)	

^a^Monthly Minimum Wage in 2015: R$788.00 = U$224.14

^b^Total varies because of missing data for each variable

^c^SUS – Unified Health System

^*P* value ≤0.1; *P value ≤0.05; **P value ≤0.001

Most of the women who attended the intervention perceived an increase in their knowledge for all three domains: 85% of evidence-based practices, 68.5% of normal birth, and 63.7% of cesarean. Women with ≤12 years of education were more likely to have perceived increased knowledge of normal birth (35.9% × 22.4% *p* < 0.001). The same was true for the perceived knowledge of EBP and cesarean. Black women were more likely to perceive an increase in knowledge for normal birth and cesarean. Women within the lower ranges of income (< 2 MW and 2 to < 5 MW) were more likely to perceive an increase in knowledge for all three knowledge domains.

Women who visited the intervention during the 1st and 2nd trimester of pregnancy were more likely to perceive an increase in knowledge for EBP (62.3% × 53.1% *p* = 0.016), normal birth (63.7% × 55.3% *p* = 0.003), and cesarean (65.6% × 52.9% *p* < 0.001). A similar trend was observed for women on their first pregnancy, with a greater proportion experiencing increases in perceived knowledge of EBP (51.3% × 36.5% *p* = 0.020) and cesarean (53.1% × 42.1% *p* < 0.001) (Table 1). Women without private health insurance were more likely to perceive an increase in knowledge for normal birth (29% × 17.1% p < 0.001).

Before the intervention, the majority of women considered themselves to have a mean score above 3.0 for all three knowledge domains. Mean scores were highest for the Normal Birth Knowledge Domain (mean = 3.71, SD = 0.94), followed by the Cesarean Knowledge Domain (mean = 3.54, SD = 0.98) and the EBP Knowledge Domain (mean = 3.15, SD = 1.08) (Table 2). In general, perceived knowledge before the intervention for each domain was higher among women who were over 35 years old, had more than 12 years of schooling, with higher income, white, with private health insurance, and in their third trimester of pregnancy.

**Table 2:** Mean knowledge about EBP, normal birth, and cesarean of pregnant women before visiting the intervention

	EBP Knowledge Domain			Normal Birth Knowledge Domain			Cesarean Knowledge Domain		
Characteristics	Mean	Std. Deviation	*P*-value	Mean	Std. Deviation	*P*-value	Mean	Std. Deviation	*P*-value
Age^c^									
19–34 years	3.12	1.079	0.037^**^	3.68	0.948	0.012^**^	3.51	0.986	0.014^**^
≥ 35 years	3.28	1.081		3.85	0.891		3.68	0.967	
TOTAL	3.15	1.081		3.71	0.940		3.54	0.985	
Education^b^									
≤ 12 years	2.48	0.888	0.000^**^	3.31	0.992	0.000^**^	2.97	0.986	0.000^**^
> 12 years	3.45	1.028		3.90	0.858		3.80	0.866	
TOTAL	3.14	1.082		3.71	0.942		3.54	0.983	
Income^a, d^									
< 2 MW	2.50	0.910	0.000^**^	3.37	0.970	0.000^**^	2.99	0.986	0.000^**^
2 to < 5 MW	3.00	1.050		3.61	0.947		3.45	0.866	
5 to < 10 MW	3.48	1.010		3.87	0.858		3.79	0.983	
≥ 10 MW	3.78	0.906		4.13	0.759		4.07	0.986	
TOTAL	3.15	1.082		3.72	0.935		3.54	0.866	
Race^b, c^									
White	3.35	1.053	0.000^**^	3.88	0.852	0.000^**^	3.76	0.890	0.000^**^
Black and Others	2.96	1.071		3.57	0.985		3.35	1.021	
TOTAL	3.14	1.080		3.71	0.939		3.54	0.985	
First Pregnancy^b^									
Yes	3.23	1.127	0.044^**^	3.74	0.975	***	3.59	1.033	***
No	3.11	1.048		3.73	0.918		3.53	0.939	
TOTAL	3.17	1.090		3.73	0.947		3.56	0.988	
Private Health Insurance^b^									
Yes	3.32	1.055	0.000^**^	3.82	0.885	0.000^**^	3.70	0.926	0.000^**^
No	2.62	0.984		3.37	1.020		3.08	1.010	
TOTAL	3.15	1.081		3.71	0.941		3.54	0.984	
Prenatal Care Coverage^b, c^									
SUS (Public)	2.96	1.110	0.000^**^	3.61	1.003	0.003^**^	3.30	1.052	0.000**
Private	3.26	1.048		3.77	0.883		3.68	0.907	
TOTAL	3.15	1.079		3.72	0.930		3.55	0.976	
Pregnancy Trimester at the time of the intervention^b, c^									
1st or 2nd	3.08	1.039	0.000^**^	3.64	0.920	0.000^**^	3.50	0.921	0.000^**^
3rd	3.36	1.097		3.91	0.899		3.72	1.003	
TOTAL	3.19	1.070		3.74	0.921		3.58	0.959	

^a^Monthly Minimum Wage in 2015: R$788.00 = U$224.14

^b^Welch Test of Equality of Means – *P*-value ≤0.05

^c^Levene Statistics – *P*-value ≥0.05

^d^Tukey Test – *P*-value ≤0.05

**P value ≤0.001

*** *P*-value was not reported since variables did not meet assumption criteria of distribution of means

**Fig. 1:**
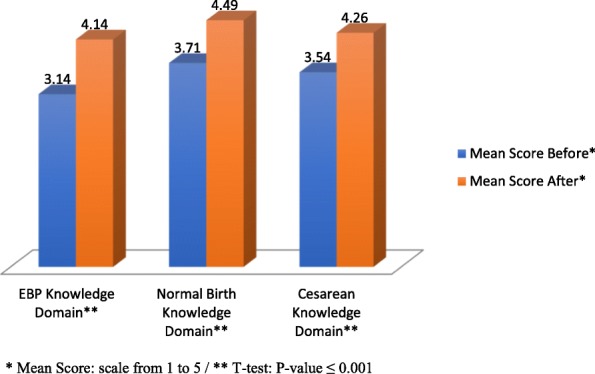
Perceived Knowledge Mean Score of EBP, normal birth and cesarean domains among pregnant women before and after the intervention

The mean score of perceived knowledge after the intervention was higher than the mean score before experiencing the SoB intervention for all three knowledge domains among pregnant women, as observed in Fig. 1. Results of the dependent (paired) sample t-tests indicated that there were significant differences in pregnant women’s perceived knowledge for evidence-based practices (*p* ≤ 0.001), normal birth (*p* ≤ 0.001), and cesarean (*p* ≤ 0.001).

Multivariate logistic regression analysis showed that women in their first pregnancy were 92% more likely to experience an increase in knowledge about EBP than women with a previous pregnancy (OR 1.92, 95% CI: 1.31–2.82); primiparous women were 37% more likely to increase knowledge about normal birth (OR 1.37, 95% CI: 1.03–1.84), and 63% more likely to increase knowledge about cesarean (OR 1.63, 95% CI: 1.23–2.16). Women in their 1st or 2nd trimester of pregnancy were also more likely than women in their third trimester to increase knowledge in all three domains (OR 1.64, 95% CI: 1.13–2.39 for EBP; OR 1.48, 95% CI: 1.11–1.97 for normal birth; OR 1.85, 95% CI: 1.40–2.41 for cesarean). Lower-income was also associated with the odds of increasing knowledge about normal birth and cesarean, but not for EBP (Table 3). Women without private health insurance (OR 2.47, 95% CI: 1.49–4.09), and those that had private prenatal care coverage (OR 2.42, 95% CI: 1.59–3.66) were more likely to experience increases in normal birth knowledge. Multicollinearity for income and education was tested, and no substantive changes in the results were observed when dropping either variable from the model.

**Table 3:** Logistic regression testing associations between socio-demographic and pregnancy information with odds of increasing perceived knowledge

	EBP Knowledge Domain^c, d^			Normal Birth Knowledge Domain^c, e^			Cesarean Knowledge Domain^c, f^		
	Total^b^*N* = 944			Total^b^*N* = 944			Total^b^*N* = 944		
Characteristics	OR	CI 95%		OR	CI 95%		OR	CI 95%	
			Wald (*P*-value)			Wald (*P*-value)			Wald (*P*-value)
Age									
19–34 years	0.895	0.564–1.419	0.223 (0.636) ^**^	1.029	0.720–1.467	0.023 (0.879) ^**^	0.925	0.653–1.311	0.191 (0.662) ^**^
≥ 35 years	1.00			1.00			1.00		
Education									
≤ 12 years	1.454	0.818–2.585	1.624 (0.202) ^**^	1.490	0.969–2.292	3.302 (0.069)^^ *^	1.359	0.911–2.027	2.254 (0.133)^^ *^
> 12 years	1.00			1.00			1.00		
Income^a^									
< 2 MW	1.888	0.865–4.112	2.546 (0.111)^^ *^	2.688	1.488–4.854	10.745 (0.001)^**^	1.499	0.864–.603	2.073 (0.150) ^** **^
2 to < 5 MW	1.687	0.966–2.946	3.377 (0.066)^^ *^	2.299	1.504–3.515	14.765 (0.000)^**^	1.757	1.161–2.660	7.115 (0.008)^* * *^
5 to < 10 MW	1.134	0.696–1.849	0.254 (0.614) ^**^	1.571	1.063–2.320	5.147 (0.023)^* *^	1.489	1.008–2.200	4.001 (0.045)^* * *^
≥ 10 MW	1.00			1.00			1.00		
Race									
Black and Others	1.099	0.747–1.617	0.229 (0.633) ^**^	0.940	0.698–1.267	0.165 (0.685) ^**^	1.192	0.895–1.596	1.401 (0.230) ^**^
White	1.00			1.00			1.00		
First Pregnancy									
Yes	1.924	1.312–2.821	11.218 (0.001)^**^	1.374	1.028–1.837	4.618 (0.032)^* *^	1.632	1.234–2.158	11.784 (0.001)^**^
No	1.00			1.00			1.00		
Private Health Insurance									
No	1.256	0.650–2.427	0.460 (0.498) ^**^	2.472	1.493–4.094	12.372 (0.000)^**^	1.303	0.818–2.076	1.245 (0.264) ^**^
Yes	1.00			1.00			1.00		
Prenatal Care Coverage									
Private	1.344	0.793–2.278	1.205 (0.272) ^**^	2.415	1.594–3.658	17.326 (0.000)^**^	1.507	1.013–2.241	4.097 (0.043)^* *^
SUS (Public)	1.00			1.00			1.00		
Pregnancy Trimester at the time of the intervention									
1st or 2nd	1.643	1.131–2.385	6.809 (0.009)^* *^	1.477	1.106–1.973	6.987 (0.008)^* *^	1.853	1.403–2.406	18.916 (0.000)^**^
3rd	1.00			1.00			1.00		

^a^Monthly Minimum Wage in 2015: R$788.00 = U$224.14

^b^Total varies because of missing data – Method of Listwise deletion

^c^Reference category: Impact on knowledge (yes)

^d^Hosmer and Lemeshow Test X^2^ = 8.451; df = 8; *P*-value = 0.391. R^2^ de Nagelkerke = 0,056

^e^Hosmer and Lemeshow Test X^2^ = 8.913; df = 8; *P*-value = 0.350. R^2^ de Nagelkerke = 0,099

^f^Hosmer and Lemeshow Test X^2^ = 11.056; df = 8; *P*-value = 0.198. R^2^ de Nagelkerke = 0,067

^*P* value ≤0.1; *P value ≤0.05; **P value ≤0.001

The linear regression model (Table 4) showed that for all three knowledge domains, women with lower income experienced greater increases in perceived knowledge than their high-income peers. For example, women with < 2 MW income experienced a greater increase in EBP knowledge than women with ≥ 10 MW by 0.206 (B = 0.206; *p* < 0.001). The same pattern was found regarding education and its association with EBP and normal birth knowledge, but not with cesarean knowledge. Greater increases in knowledge were also found to be associated with first pregnancy (for normal birth and cesarean knowledge), no private insurance, private prenatal care, and 1st or 2nd trimester at the time of the intervention.

**Table 4:** Linear regression associations between socio-demographic and pregnancy information with levels of change in perceived knowledge

	EBP Knowledge Domain^c, d^		Normal Birth Knowledge Domain^c, e^		Cesarean Knowledge Domain^c, f^	
	Total^b^*N* = 940		Total^b^*N* = 944		Total^c^*N* = 938	
Characteristics	B	*P*-value	B	*P*-value	B	*P*-value
Age						
19–34 years	−0.012	0.695	−0.011	0.729	−0.019	0.560
≥ 35 years	.		.		.	
Education						
≤ 12 years	0.142	0.000^**^	0.111	0.007^*^	0.067	0.109^^^
> 12 years	.		.		.	
Income^a^						
< 2 MW	0.206	0.000^**^	0.140	0.007^*^	0.092	0.088^^^
2 to < 5 MW	0.237	0.001^**^	0.164	0.000^**^	0.142	0.002^*^
5 to < 10 MW	0.077	0.053^*^	0.083	0.040^*^	0.098	0.018^*^
≥ 10 MW	.		.		.	
Race						
Black and Others	0.040	0.210	0.049	0.137^^^	0.079	0.010^*^
White	.		.		.	
First Pregnancy						
Yes	0.044	0.162	0.063	0.05^*^	0.096	0.021^*^
No	.		.		.	
Private Health Insurance						
No	0.154	0.000^**^	0.191	0.000^**^	0.102	0.004^*^
Yes	.		.		.	
Prenatal Care Coverage						
Private	0.146	0.001^**^	0.139	0.001^**^	0.097	0.024^*^
SUS (Public)	.		.		.	
Pregnancy Trimester at the time of the intervention						
1st or 2nd	0.100	0.001^**^	0.087	0.006^*^	0.093	0.026^*^
3rd	.		.		.	

^a^Monthly Minimum Wage in 2015: R$788.00 = U$224.14

^b^Total varies because of missing data – Method of Listwise deletion

^c^Change Score Variable - Continuous scale (−5 to 5)

^d^R-square = 0.130

^e^R-square = 0.091

^f^R-square = 0.052

*P value ≤0.05; **P value ≤0.001; ^*P* value ≤0.1

In supplemental analyses, each linear regression model was rerun after controlling for variation in the baseline perceived knowledge (before). An association between the “before perceived knowledge” and the change in knowledge was observed for all domains: EBP (B = − 0.076; *p* < 0.000), normal birth (B = − 0.729; *p* < 0.001), and cesarean (B = − 0.676; p < 0.000). Furthermore, the majority of SD and PI variables were no longer associated with increases in knowledge. The variables remaining statistically significant for the associations for each domain were the 2 to < 5 MW (B = 0.101; p < 0.000) for the EBP domain; ≤ 12 years of education (B = − 0.115; p < 0.000) and first pregnancy (B = 0.049; *p* < 0.05) for the cesarean domain.

These findings suggest that there was an increase in perceived knowledge for women with low knowledge before the intervention, regardless of their socio-demographic characteristics, prenatal care coverage, gestational age when visiting the intervention, or parity. Multicollinearity for income and education was tested, and no changes in the results were observed when dropping either variable from the model.

A summary of the results is available in Table 5.

**Table 5:** Logistic regression and linear regression summary of independent variables associated with knowledge domains

	EBP Knowledge Domain		Normal Birth Knowledge Domain		Cesarean Knowledge Domain	
	Total *N* = 940		Total *N* = 944		Total *N* = 938	
Characteristics	Logistic Regression	Linear Regression	Logistic Regression	Linear Regression	Logistic Regression	Linear Regression
Age						
19–34 years	♦	–♦	♦	♦	♦	♦
≥ 35 years	Reference		Reference		Reference	
Education						
≤ 12 years	♦	**	^	*	^	^
> 12 years	Reference		Reference		Reference	
Income^1^						
< 2 MW	^	**	**	*	♦	^
2 to < 5 MW	^	**	**	**	*	*
5 to < 10 MW	♦	*	*	**	*	*
≥ 10 MW	Reference		Reference		Reference	
Race						
Black and Others	♦	♦	♦	^	♦	*
White	Reference		Reference		Reference	
First Pregnancy						
Yes	**	♦	*	*	**	*
No	Reference		Reference		Reference	
Private Health Insurance						
No	♦	**	**	**	*	*
Yes	Reference		Reference		Reference	
Prenatal Care Coverage						
Private	♦	**	**	**	*	*
SUS (Public)	Reference		Reference		Reference	
Pregnancy Trimester at the time of the intervention						
1st or 2nd	*	**	*	*	**	*
3rd	Reference		Reference		Reference	

^*P* value ≤0.1; *P value ≤0.05; **P value ≤0.001; ♦ *P* value ≥0.1

## Discussion

The goal of this study was to evaluate the change in women’s perceived knowledge about normal birth, cesarean, and EBP after a health education intervention. Considering that the majority of the women perceived an increase in knowledge, results show that there is room to improve knowledge about normal birth, cesarean, and EBP among pregnant Brazilian women. The results also indicate that women who joined the intervention have the perceived knowledge about the risks and benefits of normal birth and cesarean above three on a scale from 1 to 5. In spite of this, women lack knowledge about how to achieve a positive childbirth experience since the majority are not aware of intrapartum EBP, and how the practices can be a tool/pathway to have a positive childbirth experience.

### The SoB intervention’s impact on women’s perceived knowledge

Most pregnant women in this study experienced an increase in perceived knowledge about normal birth, cesarean, and EBP after participating in the SoB intervention. Prior studies that evaluated the impact of a health education intervention on women’s preference, knowledge, skills, and behavior found that culturally appropriate maternity care has positive effects on increasing knowledge, stronger attitude, perceived behavior control, and the use of skilled maternity care among women [10, 64, 65]. Recent findings from the “Lancet Series of Optimizing Caesarean Section Use” also showed that non-clinical health-care interventions to reduce unnecessary C-sections are most effective when prioritizing human relationships, promoting respectful and collaborative care, and addressing women’s beliefs and attitudes [57].

The SoB intervention provides women with scientific evidence-based information, and it is a viable and valid source of women’s authoritative knowledge set, as conceptualized by different authors [44, 45]. Interactive activities through the intervention provide women with the chance to experience the embodied knowledge and the new set of authoritative knowledge gained through the visit. Increasing access to knowledge and information may empower women to challenge the authoritative and technocratic medical knowledge, providing them with the sense of self-efficacy to overcome fear, increase control, and access tools to achieve a positive experience of birth and satisfaction. Increasing women’s knowledge may also create opportunities for a meaningful conversation with their health professionals that could lead to improved support for their preference for type of birth and use of evidence-based practices to achieve a positive birth experience [20, 21, 66–68].

### Intrapartum EBP knowledge and women’s perceived control

Before the intervention, women who participated in the SoB knew more about the benefits and risks of normal birth and a cesarean than they knew about EBP. Understandably, they perceived a higher increase in their knowledge about EBP after experiencing the intervention. The lower average scores on EBP knowledge before the intervention were expected when taking into account the low rates of use of best practices among Brazilian women, shown by other studies [53, 54]. The Birth in Brazil survey found that only 3.4% of the live births between 2011 and 2012 used practices recommended by the WHO during labor and childbirth [53], and these practices were mainly used in public hospitals (SUS) and among primiparous women [38, 69].

The higher perception of knowledge for NB and Cesarean before the intervention compared to EBP knowledge might indicate that there is a broad or popular consciousness about the benefits of normal birth and risks of cesarean within Brazilian society, even while facing the technocratic model of care. However, information regarding best practices during childbirth, women’s rights, and resources to achieve a positive birth experience are not disseminated adequately. These results are consistent with the literature showing a low awareness of reproductive rights, and EBP among Brazilian women [54, 69–71].

Nonetheless, the results showed that even among highly engaged women, there is a clear need and opportunity to invest in and increase Brazilian women’s knowledge about normal birth, cesarean, and EBP during childbirth. Previous studies identified that Brazilian women had limited access to adequate information or educational practices during prenatal care, which reinforces a vertical and medical centered model of care that does not value health education as a potential quality measure/standard [70, 72].

### Creating tailored health education interventions

These results indicate that the SoB intervention was more effective for low-income women, without private health insurance, with private prenatal care, in their first pregnancy and their first or second trimester at the time of the intervention. Similar findings were observed in a study that compared two different health education interventions focused on women’s decision-making processes and preference for type of birth [73]. The results of the current evaluation point to an important group to be prioritized to optimize the potential of the intervention: primiparous, low-income women, at the beginning of pregnancy, without private health insurance. However, in Brazil, that would not include a group of women most likely to have an unnecessary cesarean, considering the existing paradox of care. White, high-income women with more than 12 years of education, with private prenatal care, are the characteristics of Brazilian women most likely to give birth in a private hospital, therefore, exposed to higher rates of C-section [36]. Therefore, there is a need to discuss how health education interventions can also be tailored to include those women who are more likely to experience a C-section in Brazil.

Different studies found that women are more likely to have a C-section if they receive private care, have limited access to midwives as primary caregivers and/or have experienced a previous cesarean [10, 74, 75]. Women with private prenatal care in this study had a higher perceived knowledge about normal birth and cesarean before the intervention than their counterparts did. However, this knowledge does not seem sufficient to impact the type of birth outcome, even though higher knowledge about the risks of C-section increases the likelihood of women waiting for labor onset and avoiding scheduling an elective surgery [76]. In the Birth Brazil Survey, 70.8% of pregnant women surveyed stated that they prefer a vaginal birth, and a meta-analysis review with 28 different Brazilian studies found an overall prevalence of 72.8% preference for a vaginal birth [37, 66]. However, 40% expected to have a C-section when arriving at the hospital to give birth [77]. Women who expect or prefer a C-section justify it by expressing their fear of fetal distress and mortality, excessive pain, or fear of trauma to the vagina [14–16, 77].

Decisions based on fear suggest a lack of knowledge or misinformation on how to have a safe birth for the mother and child, and also a society with an instilled distrust of the body’s ability to undertake labor and safely deliver a child without a negative impact on a woman’s reproductive and sexual experience. Discussing the use of EBP might be an essential aspect to achieve a positive childbirth experience, contributing to change Brazilian women’s perception of normal birth as dangerous and of cesarean as the safest type of birth. Women’s voices and values need to be incorporated into a comprehensive childbirth model to reduce unnecessary C-sections [15, 77].

Although there were no significant differences between white and black women regarding increases in knowledge, white women arrived at the intervention with higher perceived knowledge, which has also been observed in other studies about women’s decision processes regarding type of birth [73]. Considering the country’s social and racial inequalities with black women being more likely to have worse childbirth outcomes, led by social inequalities during pregnancy and throughout a lifetime exposure to discrimination and stress when accessing healthcare [78–81], discussing health education within racial differences is needed.

Given the opportunity and access to information, black women who participate in the intervention have the same chances to increase their knowledge about normal birth, cesarean and EBP as white women, which might increase their chances to achieve positive childbirth outcomes. Health education can be seen as one strategy to promote a positive childbirth experience. However, closing the black-white gap in birth outcomes will not happen without a multi-sectoral policy intervention that addresses health inequalities and systemic racism not only immediately before and during pregnancy, but also through the lens of a life-course approach [80, 82].

The observed opportunities to increase Brazilian pregnant women’s knowledge regarding all three domains (normal birth, cesarean, and EBP) can lead to questions about the quality of information women are currently receiving during prenatal care. Findings indicate that women in both the private and public health system are likely to increase their knowledge about normal birth after participating in an effective health education intervention. Meanwhile, previous non-disclosed results indicate that 16.9% of the women who had private health insurance opted for prenatal care in the public health system, as it is part of their constitutional right. Moreover, 9.5% of women who had no private health insurance opted for private prenatal care, likely paying out-of-pocket. Although not addressed here, the results point to the need of exploring quality of prenatal care in both systems, allowing women to have good quality information regardless where they are receiving it.

The current literature also supports the idea that women exposed to adequate prenatal care are more likely to increase awareness and knowledge regarding signs of obstetric dangers and benefits of normal birth and screening tests; however, few women have such access [83]. Health education and experience give mothers a more nuanced understanding of the birth process [84]. Women from different countries consider education about childbirth important and point out that it should include not only risks and benefits of the type of birth but also information about labor, delivery, and medical interventions [15, 85–88].

It is recognized that implementing scientific knowledge into practice requires a system change, and evaluating translational interventions is essential to better direct policymakers and healthcare professionals’ decisions [89]. In particular non-clinical and multicultural interventions tailored to a local context, addressing women and health professionals’ beliefs, attitudes, knowledge, and skills, as well as the limitations of the health system, are needed [57]. Therefore, health education interventions that can provide information, while promoting community engagement and giving women tools for empowerment, such as the SoB intervention, are needed to change the maternal health care scenario.

A maternal health care system as proposed by WHO to achieve SDG 3 and 5 [24] can only happen if we include women’s voices and empower them to advocate for evidence-based care, regaining control over labor and childbirth. A reproductive justice model of childbirth, focusing on building a health system that supports full reproductive health and rights, has the power to engage women in their care and reduces the impact of social inequalities on adverse birth outcomes, as demonstrated in the literature for the Zika epidemic experience [90]. In a country with high cesarean rates like Brazil, a culturally tailored intervention that can impact women’s knowledge has the potential to contribute to a maternal health care system, which includes women’s engagement and voices.

### Strengths and limitations

This is a cross-sectional study, with its intrinsic limitations that do not permit causal inferences. On the other hand, the large sample of pregnant women answering the survey allowed adequate statistical power to detect the SoB intervention’s impact on perceived knowledge about normal birth, cesarean section, and EBP to achieve a good experience in childbirth.

Brazilian women with lower perceived knowledge were likely underrepresented in our sample, which was predominantly women already sensitized about childbirth. Therefore, the impact of the SoB intervention could be underestimated. In addition, women describing their childbirth might be influenced by intrinsic social desirability, describing the birth with intense positive or negative perception. However, the anonymity and high perceived knowledge before the intervention likely diminish this influence over the results.

The SoB results gain relevance when international literature review shows a lack of evidence for the impact of non-clinical interventions to reduce unnecessary cesareans in middle and low-income countries, singularly when the interventions are focused on women [91]. In contrast, single-focus interventions that target one factor to reduce unnecessary cesareans have shown small impacts or low effectiveness [57], which might be related to the multifactorial reasons related to the TMTS and TLTL scenario.

## Conclusion

The present study indicates that participation in a culturally tailored health education intervention is associated with increases in pregnant women’s perceived knowledge of normal birth, cesarean, and evidence-based practices. Results also suggest that there are groups of Brazilian women with significantly lower knowledge regarding the included themes, which should be prioritized. However, there are opportunities to increase knowledge among all groups of women. Perceived knowledge of evidence-based practices was lower when compared with the knowledge of normal birth and cesareans, revealing a need to discuss with women how to access and use tools and resources to achieve a positive birth experience, including satisfaction with the clinical aspects of the birth process. This study reinforces that high quality tailored maternal health education can be a valuable resource to improve women’s knowledge about childbirth and reveals a need to invest in non-clinical interventions that prioritize women. Future studies, with longitudinal designs should be conducted to understand the impact of the intervention on behavior, and use of EBP, considering the type of birth and health system organization.

## Supplementary information

**Additional file 1.** Senses of Birth Pregnant Women post-test Survey.

## Data Availability

The datasets generated and/or analyzed during the current study are not publicly available due to the presence of identifiable information but are available from the corresponding author on reasonable request.

## Abbreviations

C-sections: Cesarean delivery
DF: Distrito Federal
EBP: Evidence-based Practice
IRB: Institutional Review Board
MG: Minas Gerais
MW: minimum wages
PI: pregnancy information
RJ: Rio de Janeiro
SD: social-demographic
SDG: Sustainable Development Goals
SoB: Senses of Birth
SUS: Unified Health System
TLTL: “too little, too late”
TMTS: “too much, too soon”
UN: United Nations
WHO: World Health Organization
