# Supplementary material for: Changes in perceived knowledge about childbirth among pregnant women participating in the Senses of Birth intervention in Brazil: a cross-sectional study
Source: BMC Pregnancy Childbirth. 2020 May 5;20:265. doi: 10.1186/s12884-020-02874-3 (PMC7201865; doi:10.1186/s12884-020-02874-3)
Supplement: Supplementary file 1 — Additional file 1. Senses of Birth Pregnant Women post-test Survey. [file 12884_2020_2874_MOESM1_ESM.pdf]

Senses of Birth Pregnant Women Survey

The post-test survey applied immediately after the intervention[1]

1. Date

2. Full name

3. What is your age?

4. What is your address? Neighborhood / City / State

5. What is your telephone number: Landline / Cell phone / I do not want to provide

6. What is your e-mail?

7. Marital Status: Single / Married or civil union / Widow / Separated or divorced

8. Family Income[2]

(Refer to the sum of income of all the people who live in your household)

a) Up to 1 minimum wage (<788.00)
b) From 1 to 2 minimum wages (2788.00 to 1576.00)
c) From 2 to 3 minimum wages (1576.00 to 2364.00)
d) From 3 to 5 minimum wages (2364.00 to 3,940.00)
e) From 5 to 10 minimum wages (3,940.00 to 7,880.00)
f) From 10 to 20 minimum wages (7,880.00 to 15,760.00)
g) More than 20 minimum wages (> 15,760.00)
h) I do not know / I do not want to inform

[1] Questions presented here were translated into English by the corresponding author, the original questionnaire was developed by the SOB research team and used in Portuguese

[2] Income was measured using the monthly family earning measure by the country minimum wage (< 2 minimum wages (MW), 2 to < 5 MW, and 5 to < 10 MW, and ≥10 MW). One minimum wage at the time of the intervention was approximate U$224.14, and the federal government annually defines the value.

9. Your work situation - check one option only

a) Work with a formal contract
b) Works without a formal contract
c) Public servant (municipal, state, federal or military)
d) Employee – owns the business
e) Autonomous (includes MEI)
f) Cooperative
g) I have no work that earns money
h) Other (specify)

10. What is your occupation? Check all options that apply

a) Physician
b) Nurse
c) Other health professionals (please specify)
d) Teacher
e) Middle School Student
f) High School Student
g) Undergraduate Student
h) Graduate student
i) Housewife
j) Retired
k) Other (please specify)

11. What is your schooling?

a) Incomplete middle school
b) Complete middle school
c) Incomplete high school
d) Complete high school
e) Incomplete undergrad education
f) Completed undergraduate education and more
g) I do not want to inform

12. The color of your skin is (read option)

a) White
b) Black
c) Pardo/Mulato
d) Asian/Eastern
e) Indigenous

13. Do you have private health insurance? Yes / No

14. How many times have you been pregnant before, excluding this pregnancy and counting termination (voluntary or natural)? You have had If answer = 0 move to question 17 - (Insert Number)

15. How many births were normal (including birth using forceps and vacuum)? (Insert Number)

16. Moreover, how many were cesarean? (Insert Number)

17. How many weeks/months of gestation are you?

Insert number for Weeks / Insert number for Months

18. Is your pregnancy considered high risk?

Yes / No

If yes, please describe why ___________________

19. Your prenatal appointments (current pregnancy) are covered by (check all that apply)

a) Private Health Insurance
b) SUS (National Universal Public Health System)
c) Private (direct payment to professional – out of pocket payments)
d) I never had prenatal care
e) Other (please specify)

20. Do you prefer NORMAL BIRTH?

No way / a little / Maybe / Probably / Absolutely

21. Do you prefer CESARIAN?

No way / a little / Maybe / Probably / Absolutely

22. Your knowledge about NORMAL BIRTH BEFORE the exhibition was:

None / Poor / Fair / Good / Very good

23. Your knowledge about a CESAREAN BEFORE the exhibition was:

None / Poor / Fair / Good / Very good

24. Your knowledge about NORMAL BIRTH AFTER the exhibition was:

None / Poor / Fair / Good / Very good

25. Your knowledge about a CESAREAN AFTER the exhibition was:

None / Poor / Fair / Good / Very good

26. BEFORE the exhibition, would you say your knowledge about DOULAS was:

None / Poor / Fair / Good / Very good

27. BEFORE the exhibition, would you say your knowledge about MIDWIVES/OBSTETRIC NURSES was:

None / Poor / Fair / Good / Very good

28. BEFORE the exhibition, would you say your knowledge about the Pregnant Woman's Right to have companionship, from her choice, during the labor and childbirth was:

None / Poor / Fair / Good / Very good

29. BEFORE the exhibition, would you say your knowledge about non-pharmacological birth pain relief methods was:

None / Poor / Fair / Good / Very good

30. Before the exhibition, would you say your knowledge about humanized and evidence-based care during labor and childbirth was:

None / Poor / Fair / Good / Very good

31. Before the exhibition, would you say your knowledge about organizations (NGOs, social networks, professionals, others) that defend the humanized and evidence-based care during labor and childbirth was:

None / Poor / Fair / Good / Very good

32. Before the exhibition, would you say your knowledge about the cesarean rates in Brazil was:

None / Poor / Fair / Good / Very good

33. Before the exhibition, would you say your knowledge about the Ministry of Health / World Health Organization guidelines for labor and childbirth care were:

None / Poor / Fair / Good / Very good

34. Before the exhibition, would you say your knowledge about Obstetric violence was:

None / Poor / Fair / Good / Very good

35. Before the exhibition, would you say your knowledge about Birth Plan was:

None / Poor / Fair / Good / Very good

36. Have you ever had experience with normal birth?

a) Never had normal birth
b) Positive experience
c) Negative experience

Describe __________________________

37. After the exhibition, would you say your knowledge about DOULAS is:

None / Poor / Fair / Good / Very good

38. After the exhibition, would you say your knowledge about MIDWIVES/OBSTETRIC NURSES is:

None / Poor / Fair / Good / Very good

39. After the exhibition, would you say your knowledge of the Pregnant Woman's Right to have companionship, from her choice, during the labor and childbirth is:

None / Poor / Fair / Good / Very good

40. After the exhibition, would you say your knowledge about non-pharmacological birth pain relief methods is:

None / Poor / Fair / Good / Very good

41. After the exhibition, would you say your knowledge about humanized and evidence-based care during labor and childbirth is:

None / Poor / Fair / Good / Very good

42. After the exhibition, would you say your knowledge about organizations (NGOs, social networks, professionals, others) that defend the humanized and evidence-based care during labor and childbirth is:

None / Poor / Fair / Good / Very good

43. After the exhibition, would you say your knowledge about the cesarean rates in Brazil is:

None / Poor / Fair / Good / Very good

44. After the exhibition, would you say your knowledge about the Ministry of Health / World Health Organization guidelines for labor and childbirth care are:

None / Poor / Fair / Good / Very good

45. After the exhibition, would you say your knowledge about Obstetric violence is:

None / Poor / Fair / Good / Very good

46. After the exhibition, would you say your knowledge about Birth Plan is:

None / Poor / Fair / Good / Very good

47. Your knowledge about the risks of NORMAL BIRTH BEFORE the exhibition was:

None / Poor / Fair / Good / Very good

48. Your knowledge about the risks of CESAREAN BEFORE the exhibition was:

None / Poor / Fair / Good / Very good

49. Your knowledge about the risks of NORMAL BIRTH AFTER the exhibition is:

None / Poor / Fair / Good / Very good

50. Your knowledge about the risks of CESAREAN AFTER the exhibition is:

None / Poor / Fair / Good / Very good

51. Did your preference for the type of birth (NORMAL BIRTH) change AFTER the exhibition?

No way / a little / Maybe / Probably / Absolutely

52. Did your preference for the type of birth (CESAREAN) change AFTER the exhibition?

No way / a little / Maybe / Probably / Absolutely

53. Do you think you can have a NORMAL childbirth?

No way / a little / Maybe / Probably / Absolutely

54. How will your labor and childbirth be paid? - Consider the various financing possibilities of the hospital and the professional who will attend the delivery (check all options that apply)

a) Private Health Insurance
b) SUS (National Universal Public Health System)
c) Private (direct payment to professional – out of pocket payments)
d) Private Health Insurance + out of pocket only for the obstetrician
e) Other (please specify)

55. If you had a previous cesarean section, identify the reason (s) - spontaneous response, check all options that apply.

a) I did not have a previous cesarean
b) I wanted to connect the tubes
c) I wanted to have a cesarean
d) I had one cesarean before
e) I had two or more cesareans before.
f) I did not want to feel the pain of normal childbirth
g) I fear the of lack of beds for hospitalization
h) I fear the city violence
i) My baby was wrapped in the cord
j) My baby was crossed
k) My baby was sitting
l) My baby was too big / I had no passage / I had no dilatation (failure to progress)/ my baby did not settle into my pelvis
m) I passed my due date
n) My baby was growing too slow or stopped growing
o) My placenta was old
p) My baby was suffering
q) I did not have enough amniotic liquid
r) I had a low-lying placenta
s) I had high blood pressure
t) I had diabetes
u) I had problems with HIV / AIDS
v) I had a genital ulcer/condyloma or issue in the preventive uterus cervix exam
w) I had a positive result exam for *Streptococcus* in the vagina and/or anus
x) I had a premature placental abruption
y) I had a bleeding
z) My water broke
aa) My labor never started
bb) I was pregnant with twins
cc) I had a fetal death
dd) My pregnancy induce failed
ee) I had a previous gynecologic surgery

Other (describe) ____

56. Did you have information about the benefits of normal birth during your prenatal care appointments?

Yes / No / I did not have prenatal appointment

Specify___________________________________

57. Do you participate in movement/e-mail lists/group discussions of pregnant women from humanized care to childbirth?

Yes. Specify which ____________________________________

No

Other (specify)______________________________________

58. AFTER the exhibition, have you changed your perception about the NORMAL BIRTH?

No way / a little / Maybe / Probably / Absolutely

59. After the exhibition, have you changed your perception of CESAREAN?

No way / a little / Maybe / Probably / Absolutely

60. BEFORE the exhibition you used to associate normal birth with JOY?

Never / Rarely / Occasionally / Frequently / Always

61. BEFORE the exhibition you used to associate normal birth with FEAR?

Never / Rarely / Occasionally / Frequently / Always

62. BEFORE the exhibition you used to associate normal birth with PAIN?

Never / Rarely / Occasionally / Frequently / Always

63. BEFORE the exhibition you used to associate normal birth with LOVE?

Never / Rarely / Occasionally / Frequently / Always

64. BEFORE the exhibition you used to associate normal birth with SUFFERING?

Never / Rarely / Occasionally / Frequently / Always

65. BEFORE the exhibition you used to associate normal birth with ANXIETY?

Never / Rarely / Occasionally / Frequently / Always

66. BEFORE the exhibition you used to associate normal birth with SAFETY?

Never / Rarely / Occasionally / Frequently / Always

67. BEFORE the exhibition you used to associate normal birth with CHALLENGE?

Never / Rarely / Occasionally / Frequently / Always

68. BEFORE the exhibition you used to associate normal birth with COURAGE?

Never / Rarely / Occasionally / Frequently / Always

69. BEFORE the exhibition you used to associate normal birth with RISK?

Never / Rarely / Occasionally / Frequently / Always

70. BEFORE the exhibition you used to associate normal birth with CONFIDENCE?

Never / Rarely / Occasionally / Frequently / Always

71. BEFORE the exhibition you used to associate normal birth with ACHIEVEMENT?

Never / Rarely / Occasionally / Frequently / Always

72. BEFORE the exhibition you used to associate normal birth with STRENGTH?

Never / Rarely / Occasionally / Frequently / Always

73. After the exhibition, you associate normal birth with JOY?

Never / Rarely / Occasionally / Frequently / Always

74. After the exhibition, you associate normal birth with FEAR?

Never / Rarely / Occasionally / Frequently / Always

75. After the exhibition, you associate normal birth with PAIN?

Never / Rarely / Occasionally / Frequently / Always

76. After the exhibition, you associate a normal birth with LOVE?

Never / Rarely / Occasionally / Frequently / Always

77. After the exhibition, you associate normal birth with SUFFERING?

Never / Rarely / Occasionally / Frequently / Always

78. After the exhibition, you associate normal birth with ANXIETY?

Never / Rarely / Occasionally / Frequently / Always

79. After the exhibition, you associate normal birth with SAFETY?

Never / Rarely / Occasionally / Frequently / Always

80. After the exhibition, you associate normal birth with CHALLENGE?

Never / Rarely / Occasionally / Frequently / Always

81. After the exhibition, you associate normal birth with COURAGE?

Never / Rarely / Occasionally / Frequently / Always

82. After the exhibition, you associate normal birth with RISK?

Never / Rarely / Occasionally / Frequently / Always

83. After the exhibition, you associate normal birth with CONFIDENCE?

Never / Rarely / Occasionally / Frequently / Always

84. After the exhibition, you associate normal birth with ACHIEVEMENT?

Never / Rarely / Occasionally / Frequently / Always

85. After the exhibition, you associate normal birth with STRENGTH?

Never / Rarely / Occasionally / Frequently / Always

86. What do you think influences your preference for the type of birth? Spontaneous response - Check all that apply

a) Birth stories of your family and/or your friends
b) Your husband's preference for the type of birth
c) Fear of normal birth pain
d) Fear of normal birth change your vagina
e) I wanted to bind the tubes
f) Fear of cesarean section
g) Fear of anesthesia
h) To schedule the due date
i) Have a known professional at delivery
j) Positive previous experience with normal birth
k) Previous negative experience with normal birth
l) Positive previous experience with cesarean section
m) Previous negative experience with cesarean section
n) Online information
o) Information in newspaper and magazine
p) Information on television
q) Information on pregnant women groups
r) Normal childbirth is better than cesarean section
s) Better recovery in normal birth

Other (please specify) ______________________________

87. How did you hear about the exhibit?

a) Social networks (Facebook / Instagram / Twitter)
b) Newspaper / radio / television
c) Site
d) Friend/family
e) Posters / Brochures

Other (please specify) ________

88. What brought you to the exhibition

- **a)** I was hanging around / waiting for someone/passing by
- b) I came because it is free
- c) I am interested in the subject
- d) They recommended me
- e) My institution (school/work/health center/others) has scheduled my visit

Other (please specify) _________

89. Do you usually visit exhibitions and museums?

Never / Rarely / On occasion / Frequently / Always

90. In your opinion, the exhibition was:

Bad / Regular / Good / Very good / Great

91. What did you like best about the exhibition?

- a) Gestation (baby in the belly)
- b) Surgical Maternity convenience store (product shelf)
- c) Controversies (videos with dialogues / opinions)
- d) Birth (birth tunnel)
- e) Conversations (area of texts, photos, videos)
- f) None of the option

92. Do you intend to recommend this exhibition to others?

No way / I think not / Perhaps / Most likely / Certainly.
